# Prevalence of Mental Illnesses in Domestic Violence Police Records: Text Mining Study

**DOI:** 10.2196/23725

**Published:** 2020-12-24

**Authors:** George Karystianis, Annabeth Simpson, Armita Adily, Peter Schofield, David Greenberg, Handan Wand, Goran Nenadic, Tony Butler

**Affiliations:** 1 School of Population Health University of New South Wales Sydney Australia; 2 National Drug and Alcohol Research Centre Sydney Australia; 3 Neuropsychiatry Service Hunter New England Health Newcastle Australia; 4 School of Psychiatry University of New South Wales Sydney Australia; 5 Kirby Institute University of New South Wales Sydney Australia; 6 School of Computer Science University of Manchester Manchester United Kingdom

**Keywords:** text mining, mental illnesses, domestic violence, police data, trend analysis

## Abstract

**Background:**

The New South Wales Police Force (NSWPF) records details of significant numbers of domestic violence (DV) events they attend each year as both structured quantitative data and unstructured free text. Accessing information contained in the free text such as the victim’s and persons of interest (POI's) mental health status could be useful in the better management of DV events attended by the police and thus improve health, justice, and social outcomes.

**Objective:**

The aim of this study is to present the prevalence of extracted mental illness mentions for POIs and victims in police-recorded DV events.

**Methods:**

We applied a knowledge-driven text mining method to recognize mental illness mentions for victims and POIs from police-recorded DV events.

**Results:**

In 416,441 police-recorded DV events with single POIs and single victims, we identified 64,587 events (15.51%) with at least one mental illness mention versus 4295 (1.03%) recorded in the structured fixed fields. Two-thirds (67,582/85,880, 78.69%) of mental illnesses were associated with POIs versus 21.30% (18,298/85,880) with victims; depression was the most common condition in both victims (2822/12,589, 22.42%) and POIs (7496/39,269, 19.01%). Mental illnesses were most common among POIs aged 0-14 years (623/1612, 38.65%) and in victims aged over 65 years (1227/22,873, 5.36%).

**Conclusions:**

A wealth of mental illness information exists within police-recorded DV events that can be extracted using text mining. The results showed mood-related illnesses were the most common in both victims and POIs. Further investigation is required to determine the reliability of the mental illness mentions against sources of diagnostic information.

## Introduction

Domestic violence (DV) is defined as “any incident of threatening behavior, violence or (psychological, physical, sexual, financial, emotional) abuse between adults who are or have been an intimate partner or family member, regardless of gender or sexuality” [1]. It can also occur in other relationships such as between caregivers and a dependent person (and vice versa) or those living together in a household [2]. According to the World Health Organization’s multicountry study of violence, the prevalence of physical and sexual partner violence toward women ranges from 15% to 71% globally [3,4]. In Australia, in 2018, 1 out of 6 women and 1 out of 16 men experienced physical or sexual violence or both by a current or previous partner [5] and on average, 1 woman a week is murdered by her current/former partner [6]. In addition, research has shown that children exposed to DV experience long-term effects on their development with increased risk of mental health issues, learning difficulties, and behavioral problems [7]. DV puts significant economic and health burden on the community and its prevention should be a public health priority [8]. Estimates have suggested that the annual financial burden in Australia arising from DV against women and their children is over AUD 22 billion (~US $16.3 billion), £66 billion (~US $89 billion) in the United Kingdom, and US $55 billion in the United States [9-11].

DV has been linked to significant comorbidity and mortality with both short- and long-term health consequences, particularly among women [3,8,12]. Evidence has shown that any immediate injury or trauma suffered in a DV setting has longer-term negative effects on the survivor’s well-being, contributing to poor health outcomes including post-traumatic stress disorder, chronic substance use, risky sexual behaviors, eating disorders, suicidal tendencies and attempts, as well as exacerbation of psychotic symptoms [3,4,12].

Associations have been found between mental health conditions (eg, bipolar disorder, schizophrenia) and the perpetration of violence toward others fueling perceptions that label these individuals as *dangerous*, leading to the stigmatization of this group [13-19]. Increasing evidence suggests that people with mental illness and psychiatric symptoms, however, are at a greater risk of victimization when compared to those without such symptoms [3,8,12,13,20-22]. Women with disabilities, including those with chronic mental or emotional conditions, experience higher rates of violent victimization than men with disabilities and women in the general population [23]. Men and women with severe mental illness (such as psychotic disorders) are two to eight times more likely to experience any form of DV abuse and to suffer poor health outcomes (eg, suicide attempt, substance abuse) than the general population [20,24]. This suggests the potential importance of knowing whether an individual has a pre-existing mental illness at the time of a DV event to enable prevention and intervention measures to be enacted.

The New South Wales Police Force (NSWPF) attends and subsequently records thousands of DV events each year—123,330 such events were recorded in 2017—in free text in their WebCOPS database, an online interface for the Computerized Operational Policing System (COPS) that enables the police to capture and analyze crime information on an organization-wide basis (NSWPF, personal communication). These police-recorded DV events contain a wealth of unutilized mental illness information for persons of interest (POIs)—individuals involved in a DV event that have been accused or charged for perpetrating DV related crimes—and victims that could be used to identify trends in those involved in DV and assist in shaping early DV intervention and prevention policies. However, the vast number of such events make the manual extraction of potentially useful information with traditional ethnographic/qualitative approaches impractical. Indeed, one recent research paper commented that “…there is no systematic way to extract information from these [police] narratives other than by manual review” [25].

Automated methods for large-scale processing of free text known as text mining have been used for over 30 years to harvest information from unstructured text in many domains, including medicine [26,27]. Several attempts have been made to extract mental health–related information from various free-text resources [28-33] including identification of drug side effects from psychiatric narratives by applying rule- and dictionary-based methods and machine learning approaches [28,30,31]. There have been efforts to extract treatment outcomes for major depressive disorders from electronic medical records with a supervised approach combined with logistic regression [29], whereas Jackson et al [33] and Karystianis et al [32] identified psychiatric symptoms from clinical discharge summaries and psychiatric records using regular expression pattern matching and a rule-based approach, respectively. Most recently, Wu et al [34] applied dictionary and machine learning methods to extract depressive symptoms in order to validate the diagnosis of major depressive disorders from electronic health records.

Because of the rapid implementation of automated technologies in various fields, text mining has been identified as a potential tool of interest in the analysis of police data. However, there are relatively few text mining methods that have been developed to analyze police narratives. Recent work has been conducted in automatically processing police reports to identify information of interest [35-38]. Attempts have been made to automatically identify offenders’ names, illicit drugs, and weapons with various degrees of success from police narrative reports through named entity extractors [35,36], while others aimed to classify police reports as DV or non-DV related using an unsupervised clustering method [37]. Most recently, deep learning methods have been used to extract mental health–related incidents from police narratives with an 89% accuracy [38].

This study builds on our previously published work that focused solely on the design, description, and evaluation of the text mining methodology [39]. We present the extracted mental illness mentions from 416,441 police-recorded DV events that involve single POIs and victims by age groups and sex, and compare the prevalence of the identified information with a fixed field mental illness flag also recorded in the WebCOPS system for the same cohort, as well as with the national estimated prevalence of mental illness in Australia. To the best of our knowledge, this is the first attempt to report automatically extracted mental health information from a large cohort of police-recorded DV events.

## Methods

### Data

Information relating to DV events that the police attend is recorded in their WebCOPS database as both structured data (fixed fields) covering demographic information (eg, name, date of birth, Aboriginal status, whether weapons were used) and free-text *event narratives*. Each police-recorded DV event contains at least one event narrative which details the incident(s) between the POI and victim, covering the circumstances of the event, whether alcohol, drugs, or both were involved, and any action(s) taken by the police. The text narratives can contain misspellings and typographical errors, often with informal acronyms, jargon, and abbreviations, that may bear ambiguous meanings depending on the context. Typically, they are used as an aide-memoire for the police and lawyers should the case proceed to court and by lawyers in court proceedings, but they have not been utilized in a substantive manner for research purposes.

We obtained 492,393 police-recorded DV events from the NSWPF from January 2005 to December 2016 that were flagged in the fixed fields with one of the following tags: “domestic” as the type of offence, “domestic violence related” as the associated factor of the police event, or the relationship status between the victim and the POI being described as spouse/partner (including ex-spouse/ex-partner), boy/girlfriend (including ex-boy/ex-girlfriend), parent/guardian (including step/foster), child (including step/foster), sibling, other member of family (including kin), or carer. These police-recorded DV events covered the following incident categories: assaults, breaches of Apprehended Violence Orders, homicides, malicious damage to property, and offences against another person such as intimidation, kidnapping, abduction, or harassment. The police-recorded DV events also contained incidents where no crime was committed but the police did attend and record the event. A hypothetical deidentified police-recorded DV event is shown in Multimedia Appendix 1.

### Ethics

Permission to access the police-recorded DV events was granted by the NSWPF following ethics approval from the University of NSW Human Research Ethics Committee (HC16558).

### Extraction and Normalization of Mental Illness Mentions

We designed and applied a text mining methodology that was implemented through the General Architecture for Text Engineering (GATE; 8.4.1 version) [40], a text mining framework to capture mental illness mentions (including traumatic brain injury and dementia) for POIs and victims. GATE was selected because it supports the development of rule-based approaches as it can easily manipulate unstructured data. We developed 2 sets of rules: based on common lexical patterns observed in the text of 200 police-recorded DV events that indicate the presence of a specific mental health mention for a POI (eg, “POI is suffering from dementia”) or for a victim (eg, “the victim was diagnosed with paranoid schizophrenia”) and based on related semantic anchors (eg, “POI,” “defendant” for POIs and “victim,” and “vic” for victim) including cases where:

- unspecified mental disorders were recorded simply as “the defendant has *mental health issues*,” “victim is suffering from a *severe mental disorder*”;
- psychotropic drugs were used by the POI or victim (eg, “the victim takes *Valium*,” “accused takes a number of *antidepressants*”) that might indicate a mental illness categorized in 4 groups (antianxiety, antidepressants, neuroleptics, antipsychotics);
- individual had traumatic brain injury, drug prescription abuse (unspecified in the text regarding the medication), substance abuse (unspecified in the text regarding the substance), and drug-induced disorders (unspecified in the text regarding the drug; see Multimedia Appendix 2 for the full reference list including our own 8 categories).

These rules were combined with dictionaries of terms for mental illness including common abbreviations and synonyms. The methodology was fully evaluated against the manual annotations of mental illness mentions for POIs and victims by 2 experts (in DV and neuropsychiatry, respectively) in a random sample of 100 police-recorded DV events, and returned an average 92% precision (ie, the percentage of correctly identified mental illness mentions against the total number of identified mental illness mentions, a denominator that includes both true-positive and false-positive mentions of mental illness by text mining) for the extracted mental illness mentions for both POIs (97.5% precision) and victims (87.1% precision); a detailed description has been published elsewhere [39].

The extracted mental illness mentions based on the description provided in the police records ranged from general descriptions (eg, “mood disorder,” “behavioral problems”) to very specific mentions (eg, “oppositional defiance disorder,” “paranoid schizophrenia”). To impose a suitable structure for further analysis of the mental health data, we mapped the mental illness mentions to the World Health Organization’s International Classification of Diseases (ICD-10) Mental and Behavioural Disorders categories using 4 levels (Multimedia Appendix 3) [41]. We utilized the expertise of the fourth author (PS) in the field of neuropsychiatry in cases where the mapping was ambiguous. The first level of mapping included 18 categories based on the ICD-10 with 8 additional customized categories; 4 categories where no specific disorder was mentioned but mental illness was implied by mention of a particular medication (see 22-25, Multimedia Appendix 2). This included specific drug classes such as antidepressants or specific brand names such as Zoloft. Four additional categories were added covering “drug prescription abuse,” “substance abuse (unspecified),” “traumatic brain injury,” and “unspecified drug induced disorders.” Cases in which we recognized that either the victim or the POI had an unknown mental illness, or an unknown drug-induced mental disorder, were assigned into the categories of “unspecified mental disorder” and “unspecified drug induced disorder,” respectively.

Cases in which mental illness mentions were more specific were mapped to lower-level ICD-10 categories. For example, the mention of “acute stress reaction” was classified at the third level according to the ICD-10 schema. Because the mention had a third-level mapping, this indicates that it can also be mapped upward to the second ICD-10 level (Reaction to severe stress and adjustment disorders) and first ICD-10 level, respectively (Anxiety, dissociative, stress-related, somatoform, and other nonpsychotic mental disorders).

In some narratives, a fourth level of detail based on the ICD-10 classification containing 27 categories was recorded by the police. However, for the purpose of reporting in this paper, we combined the fourth and third levels, for example, instead of reporting “other impulse disorders” (third level), we included “intermittent explosive disorder” (fourth level) in the third classification level (Multimedia Appendix 3 shows some mapped examples of extracted mental illness to ICD-10). For reporting purposes, we show only police-recorded DV events that involved POIs and victims with mental illness at the second level of ICD-10 because the first-level ICD-10 descriptors are too broad (eg, mood [affective] disorders).

Despite utilizing 2 sets of rules that correctly identified whether a mental illness mention was linked to a POI or a victim within a DV event, this text mining methodology was unable to associate the extracted mental illness “mention” with a specific POI or victim, if more than 2 individual POIs or victims were present. Thus, we focused only on those DV events that included a single POI and a single victim which included a total of 416,441 DV events out of 492,393. In this analysis results are presented for 416,441 police-recorded DV events. Seven age groups were defined (0-14 years, 15-24 years, 25-34 years, 35-44 years, 45-54 years, 55-64 years, and 65 years and over) to align with the national reporting practices of the Australian Institute of Health and Welfare’s Family, Domestic and Sexual Violence in Australia [42].

## Results

### Study Analysis

Overall, 15.51% of police-recorded DV events (64,587/416,441) had at least one mention of a mental illness for either the POI, victim, or both. In almost three-quarters (49,154/64,587, 76.11%) of these events, there was only a mental illness mention for the POI, 17.29% (11,167/64,587) for the victim, and 6.61% (4266/64,587) for both the victim and POI (Table 1). The total number of mental illness mentions classified at level 1 was 85,880 (100%); level 2, 51,858/85,880 (60.38%); and level 3, 26,704/85,880 (31.09%; Table 1). It should be noted that 1 police-recorded DV event can have more than 1 (different) mental illness mentions associated with a POI or victim. This can be seen in Table 1, where the total number of mental illness mentions is greater than the number of police-recorded DV events with a mental illness for a POI or a victim.

**Table 1:** Numbers of police-recorded DV events with mental illness mentions for POIs and victims (first column), and numbers of the mental illness for POIs and victims at various levels of the International Classification of Diseases-10 groupings.

Individual type	Police-recorded DV^a^ events, n	Mental illness mentions (third level), n	Mental illness mentions (second level), n	Mental illness mentions (first level), n
POI^b^ only	49,154	20,452	39,269	67,582
Victim only	11,167	6252	12,589	18,298
POI and victim	4266	N/A^c^	N/A	N/A
Total	64,587	26,704	51,858	85,880

^a^DV: domestic violence.

^b^POI: persons of interest.

^c^N/A: not applicable.

As police data are collected at the police-recorded DV event level, a single victim or POI may have multiple events over time. Of the 416,441 police-recorded events, the total number of unique POIs with a recorded mental illness was 18.53% (39,688/214,185) with 5.61% (13,709/244,219) for unique victims. This was lower than the current estimated prevalence from the Australian Bureau of Statistics for 2017-18, which revealed that 20.1% of the Australian population had a current mental or behavioral condition [43].

**Figure 1:**
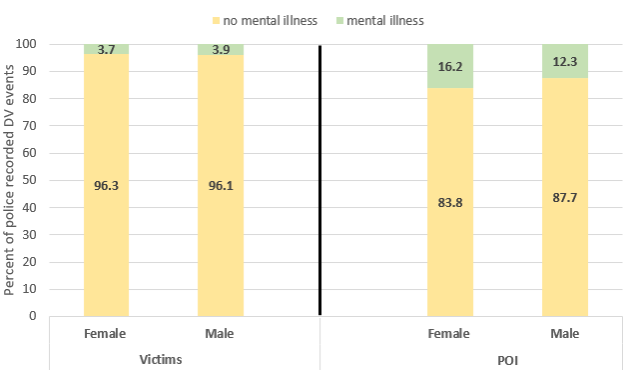
Proportion of police-recorded DV events with mental illness by sex of victims and POIs.

The percentage of police-recorded DV events in which victims had a mention of mental illness was lower than for POIs for both women and men (3.70% [11,523/311,210] and 3.86% [3718/96,228] vs 16.21% [12,048/74,323] and 12.30% [40,514/329,306]; Figure 1). Interestingly, 16.21% (12,048/74,323) of police-recorded DV events with female POIs had a recorded mental illness compared with 12.30% for men (40,514/329,306; Figure 1).

It is important to present “sampling errors” associated with the point estimates using confidence intervals. However, in our study 95% CIs were extremely narrow due to the large sample sizes. For example, the point estimate for the proportion of mental illness among female victims was 3.70% (11,523/311,210) with 95% CI of 3.6%-3.8%, indicating that 3.70% (11,523/311,210) was estimated with less than ±0.1% precision. Similarly, the proportion of mental illness among male victims was 3.86% (3718/96,228) with 95% CI of 3.7-4.0 (ie, 3.86% [3718/96,228] was estimated with less than ±0.1% precision). Extremely narrow intervals were also observed for the POIs—proportion of mental illness for females: 16.21% (12,048/74,323) with 95% CI of 15.9%-16.5%; proportion of mental illness for males: 12.30% (40,514/329,306) with 95% CI of 12.2-12.4.

**Figure 2:**
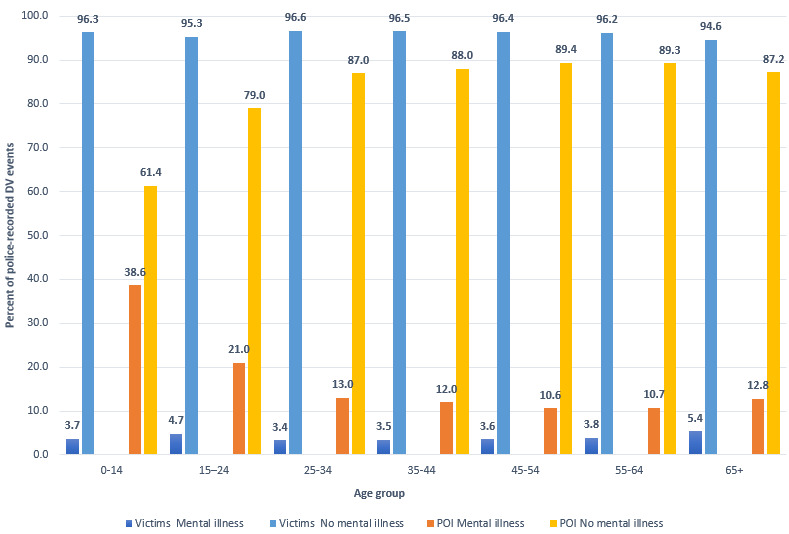
Percentage of police-recorded DV events with POIs and victims with or without a mental illness by age group.

When looking at the proportion of police-recorded DV events with mental illness by age group, the highest proportion of police-recorded DV events with a mental illness among victims was for the 65 years and over age group (1227/22,873, 5.36%; Figure 2). However, for POIs, the youngest age group showed the highest proportion of police-recorded DV events with a mental illness (623/1612, 38.64%, for POIs aged 0-14 years; Figure 2).

### Mental Illness Reported in ICD Levels 1 and 2

At the first ICD classification level (Table 2), almost one-third of the mentions of mental illness (22,172/67,582, 32.81%) for POIs and one-fifth (4208/18,298, 23.00%) for victims were classified as “unspecified mental illness,” meaning the type of mental illness was not explicitly recorded in the narratives by the attending police officer(s). “Mood [affective] disorders” (eg, bipolar disorder, depression) had the highest number of mentions among POIs (12,753/67,582, 18.87%) and victims (4288/18,298, 23.43%), with “mental and behavioral disorders due to psychoactive substance use” (including alcohol abuse) ranking fourth and fifth for POIs (5642/67,582, 8.35%) and victims (1098/18,298, 6.00%), respectively. For POIs, 11.45% (7735/67,582) had mentions of “behavioral and emotional disorders with their onset usually occurring in childhood and adolescence” (eg, “attention deficit hyperactivity disorders,” “conduct disorders”), whereas for victims this was 9.77% (1787/18,298). Although mentions of intellectual disabilities among POIs (n=1276) were higher than in the victims (n=813), as a percentage (of police-recorded DV events among POIs and among victims separately) this proportion was higher in victims (813/18,298, 4.44%) than POIs (1276/67,582, 1.89%). Mentions of traumatic brain injury (eg, “the victim has suffered a brain injury due to a car accident”) were reported for 0.84% of POIs and 1.10% victims (568/67,582 and 201/18,298 mentions, respectively).

In the second-level categories (Table 3), “major depressive disorder, single episode” was the most commonly reported mental illness for both victims (2822/12,589, 22.42%) and POIs (7496/39,269, 19.01%), while alcohol abuse was the second highest mental illness among POIs (4867/39,269, 12.39%) and the fifth highest reported among victims (1033/12,589, 8.21%), further supporting the association between DV and alcohol use [44]. Additionally, there were 576 police-recorded DV events with victims (576/12,589, 4.58%) and 486 police-recorded DV events with POIs (486/39,269, 1.24%) with dementia. At the third level, “bipolar disorder, unspecified” was the most prevalent for both POIs (4462/20,452, 22.82%) and victims (1362/6252, 21.79%) with similar rates, followed by “unspecified behavioral and emotional disorders” (3930/20,452, 19.22%) for POIs and “anxiety disorder, unspecified” (1274/6252, 20.38%) for victims.

**Table 2:** Number of police-recorded DV events (%) with a mental illness mention grouped by the first ICD-10 level for POIs and victims from 416,441 DV events recorded by the New South Wales Police Force in Australia between 2005 and 2016.

Mental illness (ICD-10^a^ code)	Police-recorded DV^b^ event frequency (POI^c^), n (%) (N=67,582)	Prevalence, %^d^	Police-recorded DV event frequency (victim), n (%) (N=18,298)	Prevalence, %^d^
Unspecified mental disorder (F99)	22,172 (32.81)	5.32	4208 (23.00)	1.01
Mood (affective) disorders (F30-39)	12,753 (18.87)	3.0.6	4288 (23.43)	1.02
Behavioral and emotional disorders with onset usually occurring in childhood and adolescence (F90-99)	7735 (11.45)	1.85	1787 (9.77)	0.42
Mental and behavioral disorders due to psychoactive substance use (F10-19)	5642 (8.35)	1.35	1098 (6.00)	0.2.6
Schizophrenia, schizotypal, delusional, and other nonmood psychotic disorders (F20-29)	4751 (7.03)	1.14	893 (4.88)	0.21
Anxiety, dissociative, stress related, somatoform, and other nonpsychotic mental disorders (F40-49)	3034 (4.49)	0.72	1961 (10.72)	0.47
Intentional self-harm (X71-83)	2702 (4.00)	0.64	821 (4.49)	0.19
Substance abuse	2310 (3.42)	0.55	314 (1.72)	<0.1
Pervasive and specific developmental disorders (F80-89)	1492 (2.21)	0.35	417 (2.28)	0.10
Intellectual disability (F70-79)	1276 (1.89)	0.30	813 (4.44)	0.19
Disorders of adult personality and behavior (F60-69)	1096 (1.62)	0.26	369 (2.02)	<0.1
Injury of unspecified body region (T14)	687 (1.02)	0.16	221 (1.21)	<0.1
Traumatic brain injury	568 (0.84)	0.13	201 (1.10)	<0.1
Mental disorders due to known physiological conditions (F01-09)	498 (0.74)	0.11	580 (3.17)	0.13
Medication—antidepressants	326 (0.48)	<0.1	114 (0.62)	<0.1
Symptoms and signs involving cognition, perception, emotional state, and behavior (R40-46)	168 (0.25)	<0.1	74 (0.40)	<0.1
Medications—antipsychotics	108 (0.16)	<0.1	13 (0.07)	<0.1
Medications—antianxiety	77 (0.11)	<0.1	20 (0.11)	<0.1
Other degenerative diseases of the nervous system (G30-32)	54 (0.08)	<0.1	47 (0.26)	<0.1
Chromosomal abnormalities, not elsewhere classified (Q90-99)	48 (0.07)	<0.1	32 (0.17)	<0.1
Unspecified drug-induced disorders	43 (0.06)	<0.1	0 (0.00)	<0.1
Behavioral syndromes associated with physiological disturbances and physical factors (F50-59)	24 (0.04)	<0.1	18 (0.10)	<0.1
Systematic atrophies primarily affecting the central nervous system (G10-14)	11 (0.02)	<0.1	6 (<0.03)	<0.1
Diseases of the nervous system	3 (<0.01)	<0.1	3 (<0.01)	<0.1
Drug prescription abuse	3 (<0.01)	<0.1	0 (<0.01)	<0.1
Medication—neuroleptics	1 (<0.01)	<0.1	0 (<0.01)	<0.1

^a^ICD: International Classification of Diseases.

^b^DV: domestic violence.

^c^POI: persons of interest.

^d^Prevalence was calculated by dividing the number of police-recorded DV events with an ICD level 1 mention with the total number of police-recorded DV events.

**Table 3:** The 20 most commonly reported mental illnesses for both POIs and victims (at the second and third level of the ICD-10 categories) in 416,441 DV events recorded by the New South Wales Police Force in Australia between 2005 and 2016.

ICD-10^a^ level 2			ICD-10 level 3			
Mental illness (ICD-10 code)	Police-recorded DV^b^ events (POI^c^), n	Police-recorded DV events (victim), n		Mental illness (ICD-10 code)	Police-recorded DV events (POI), n	Police-recorded DV events (victim), n
Major depressive disorder, single episode (F32)	7496	2822		Bipolar disorder, unspecified (F31.9)	4462	1362
Alcohol abuse (F10)	4867	1033		Unspecified behavioral and emotional disorders with onset usually occurring in childhood and adolescence (F98)	3930	636
Bipolar disorder (F31)	4487	1361		Schizophrenia, unspecified (F20.9)	3762	711
Schizophrenia (F20)	4032	732		Anxiety disorder, unspecified (F41.9)	1910	1274
Other behavioral and emotional disorders with onset usually occurring in childhood and adolescence (F98)	3939	636		Autism (F84.0)	768	277
Attention deficit hyperactivity disorder (F90)	3045	1041		Suicide attempt (T14.91)	686	221
Other anxiety disorders (F41)	2001	1494		Cyclothymic disorder (F34.0)	633	71
Pervasive developmental disorder (F84)	1444	394		Post-traumatic stress disorder (F43.1)	619	327
Specific personality disorders (F60)	1074	329		Oppositional defiant disorder (F91.3)	614	92
Intellectual disability, unspecified (F79)	1023	669		Asperger syndrome (F84.5)	588	117
Conduct disorders (F91)	687	99		Paranoid personality disorder (F60.0)	538	137
Injury of unspecified body region (T14)	687	221		Personality disorder, unspecified (F60.9)	236	88
Persistent mood disorder (F34)	641	71		Obsessive compulsive disorder, unspecified (F42.9)	234	66
Reaction to severe stress, and adjustment disorders (F43)	638	335		Postpartum depression (F32.9)	227	233
Unspecified psychosis not due to a substance or known physiological condition (F29)	512	104		Borderline personality disorder (F0.3)	219	87
Dementia, unspecified (F03)	486	576		Paranoid schizophrenia^d^ (F20.0)	207	23
Other psychoactive substance-related disorders^d^ (F19)	291	19		Suicidal ideations (R45.85)	161	74
Obsessive-compulsive disorder (F42)	234	66		Dissociative identity disorder (F44.81)	114	38
Other stimulant related disorders^d^ (F15)	213	16		Panic disorder (F41.0)	85	218
Cannabis abuse^d^ (FF12)	174	12		Conduct disorder, unspecified^d^ (F91.9)	72	7
Intellectual disability, mild^e^ (F70)	133	73		Alzheimer disease, unspecified^e^ (G30.9)	46	46
Symptoms and signs involving emotional state^e^ (R45)	168	72		Down syndrome, unspecified^e^ (Q90.9)	48	32

^a^ICD: International Classification of Diseases.

^b^DV: domestic violence.

^c^POI: persons of interest.

^d^Top 20 common mental illnesses—POIs only.

^e^Top 20 common mental illnesses—victims only.

### Mental Illness by Age

#### Persons of Interest

**Figure 3:**
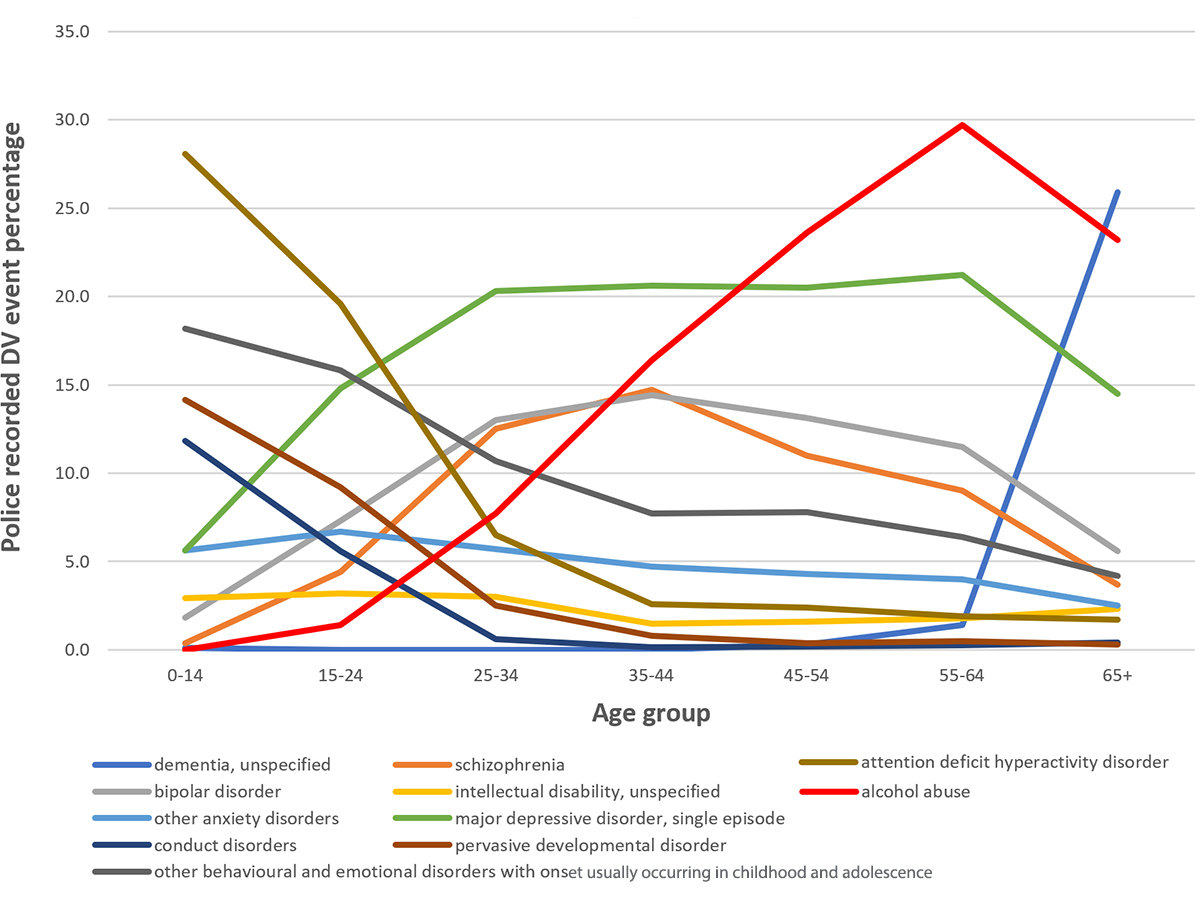
Police-recorded DV event percentages for the top ten commonly reported mental illnesses across age groups for POIs.

The proportion of police-recorded DV events for POIs, “alcohol abuse” showed an increase from 15-24 years across all older age groups with the highest proportion in the 55-64 years group (695/2340, 29.70%; Figure 3). The most commonly reported mental illness for those who were 65 years and over was “dementia, unspecified” (320/1235, 25.91%).

By contrast, “attention deficit hyperactivity disorder” was the most prevalent mental illness among the younger age groups making up 28.1% (230/818) and 19.60% (1657/8454) of police-recorded DV events for the 0-14 years and 15-24 years age groups, respectively. Mental illness associated with younger populations such as “pervasive development disorder” and “conduct disorders” made up a relatively high proportion of police-recorded DV events for their age groups (116/816 [14.2%] and 97/822 [11.8%], respectively). Mental illness in the younger age groups showed a decline with increasing age (eg, “pervasive developmental disorders,” “conduct disorders,” “attention deficit hyperactivity disorder,” “other behavioral and emotional disorders with onset during childhood,” “other anxiety disorders,” and “intellectual disability, unspecified”).

“Major depressive disorder, single episode” was the most common mental illness in police-recorded DV events involving those aged 25-34 years and 35-44 years (1927/9492 [20.30%] and 1880/9126 [20.60%], respectively). “Bipolar disorder” and “schizophrenia” showed a similar trend, increasing as a proportion of their age group, until the 35-44 years age group, and then gradually decreasing for the older age groups.

#### Victims

**Figure 4:**
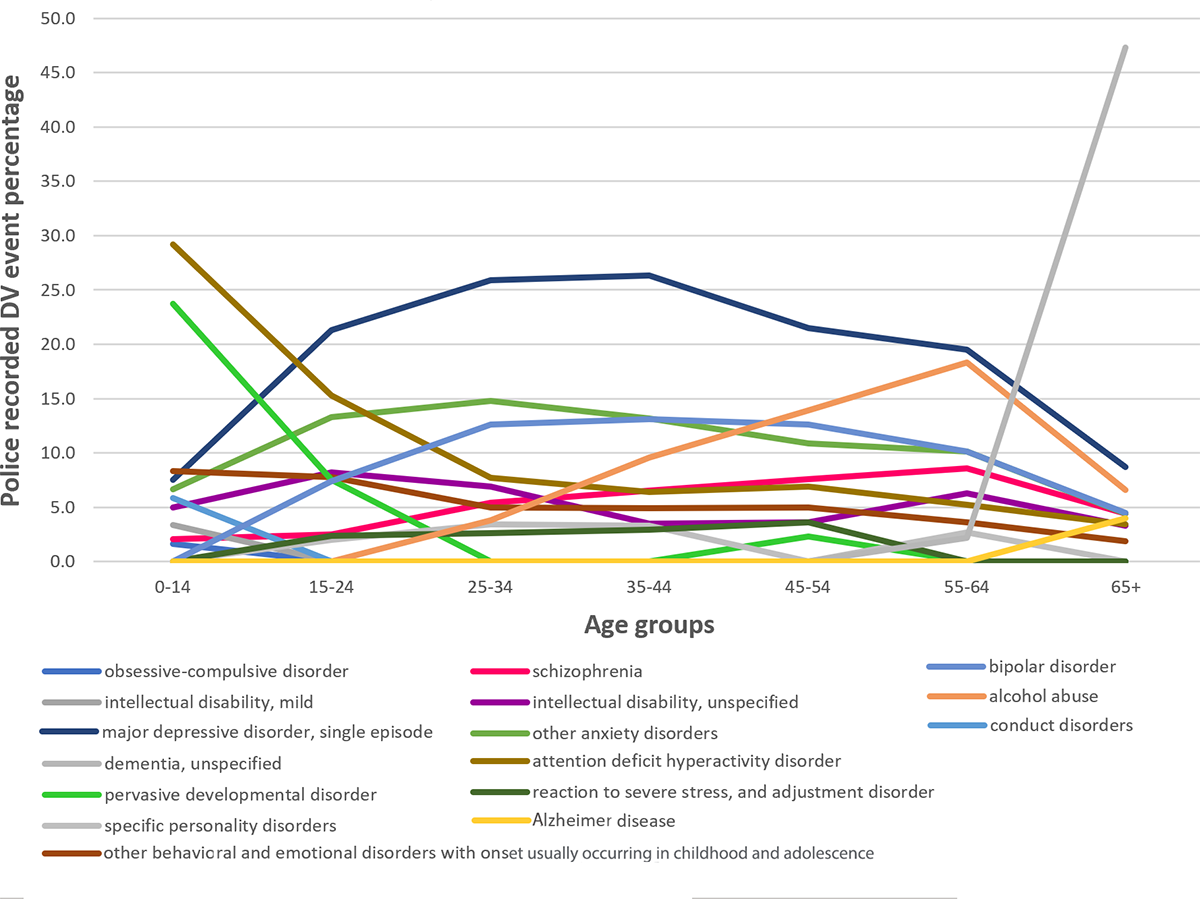
Police-recorded DV event percentages for the top ten commonly reported mental illnesses across age groups for victims.

Excluding the youngest and oldest age groups (ie, 0-14 and 65 years and over), “major depressive disorder, single episode” made up the greatest proportion of recorded mental illness across all other age groups ranging from 19.51% (224/1148; 55-64 years) to 26.30% (748/2844; 35-44 years). “Attention deficit hyperactivity disorder” made up 29.1% (70/240) of recorded mental illnesses for those aged 0-14 years and for victims aged 65 years and over, “dementia, unspecified” was recorded for almost half (515/1088, 47.33%) of police-recorded DV events with a reported mental illness (Figure 4).

Conditions in the younger groups (0-14 and 15-24) such as “pervasive developmental disorder” and “conduct disorders” gradually decreased across older age groups. As a proportion of recorded mental illnesses, “major depressive disorder, single episode” and “bipolar disorder” increased from the younger age groups to their highest in the 35-44 year group (748/2844 [26.30%] and 374/2854 [13.10%], respectively), and then steadily decreased in the older age groups (95/1091 [8.71%] for “major depressive disorder, single episode” and 50/1111 [4.50%] for “bipolar disorder” in the 65 years and over old group). Additionally, “alcohol abuse” showed a steady increase across the age groups reaching a peak in the 55-64 years age group (210/1147, 18.31%). A similar trend was observed for “schizophrenia,” increasing to 8.71% (99/1137) of police-recorded DV events with a mental illness mention for the 55-64-year group. For the exact percentages for Figures 3 and 4, see Multimedia Appendices 4 and 5.

## Discussion

### Principal Findings

By text mining a large, population-based data set of DV events recorded by the NSWPF, our findings indicate that a large number of those events (64,587/416,441, 15.51%) involve individuals (victims and POIs) who may have a mental illness. The findings are important in raising awareness about the significance of mental illness in the context of DV and have implications for the training of front-line police officers in managing those with mental illness attending DV events. While there is a growing literature concerned with the association between mental illness and DV, no other published study, as far as we are aware, has reported on the real-time capture of mental illness data by front-line police officers attending and recording DV events.

These findings complement previously published research indicating that mental illness can increase the risk of being in an abusive relationship, as either a POI or victim (or both) [3,14,20,45]. Further research is required to explore the unique context in which this arm (the police) of the justice system interacts with individuals with mental illness and how this can be optimized to improve outcomes in these situations. Police officer detection of possibly relevant mental illnesses that are virtually contemporaneous when attending to a DV event can lead to the identification of potential new strategies and interventions to tackle this issue. An example could be the development of a mobile app utilized by the police who by recording a mental health diagnosis can receive guidelines to de-escalate the DV situation or improve their decision making.

Our results showed that the prevalence of mental illness for unique victims (13,709/244,219, 5.61%) and POIs (39,688/214,185, 18.53%), respectively, was lower than that of the estimated national prevalence of mental illness reported in the 2017-18 National Health Survey (20.1%, 4.8 million) [43]. There are several possible reasons as to why our estimated prevalence was lower. Our data were from 2005 to 2016, and rates of mental illness may have increased over time. The National Health Survey sets out to systematically capture prevalent mental illness. By contrast, underreporting of mental illness to the police in these events is to be anticipated given that the detection and recording of mental health status are not the focus of the police’s work since their role is not to diagnose or inquire about mental illness but to prioritize victim safety and diffuse the situation. Most likely, mental illness is reported in a very ad hoc manner and only if the POI, victim, or other person divulges this information.

Our estimates of mental illness were derived from unique persons from single victim to single POI police-recorded DV event data, while police-recorded DV events with multiple POIs or victims were excluded (and therefore some mental illness-related information was lost). The National Health Survey’s self-reported mental and behavioral conditions information may encompass a broader definition of mental health and well-being than what was captured in the police narratives [43]. It is noteworthy that the difference in the overall rates of mental illness mentions found in this study—lower in victims than in POIs—does not seem to be reflected in the existing literature. It is possible that a bias exists in the context of police questioning on the mental health status of the POIs such that relatively more mental illnesses are reported for this group. Such a bias, if confirmed, would have potentially important implications, particularly if the detection of mental illness by police were to influence the provision of immediate support. Finally, false negatives (correct mentions of mental illness for POIs and victims ignored by the rules) generated by the application of text mining could potentially have contributed to this low prevalence, prompting to cast a wider net of rules that could capture more generic mentions and avoid the reliance on semantic anchors.

We found differences in the top 10 most commonly reported mental illnesses across age groups for POIs and victims. While the top 10 most reported mental illnesses among POIs remained consistent across age groups, this did not occur for victims. For example, “obsessive compulsive disorder” and “intellectual disability, mild” were only reported once among younger age groups. “Alzheimer disease, unspecified” was one of the most commonly reported illnesses for the 65 years and over age group for victims.

Given the low representation of personality disorder in our data, while this being a common diagnosis for DV perpetrators in published studies, it is plausible that personality disorder might account for a significant proportion of unspecified mental illness, and more so in perpetrators than victims [46].

Studies have consistently found heavy alcohol use to be associated with DV in both men and women [47,48]. In NSW, the Australian Institute of Criminology has shown evidence for alcohol misuse as an important risk factor for DV [49]. Our results indicated that the number of police-recorded DV events among POIs involving “alcohol abuse” increased with increasing age, with the highest number of police-recorded DV events shown to be among POIs of 55-64 years old. These findings, at population-level sample, support the link between alcohol abuse and DV.

Conditions that (usually) occur in childhood such as “attention deficit hyperactivity disorder” or “conduct disorders” were understandably most prevalent in the younger age groups. Individuals with these conditions are potentially vulnerable to domestic abuse [50] as well as at risk of committing violence toward parents, peers, or carers, likely reflecting the fact that impaired behavioral self-regulation implied by these diagnoses serves as a risk factor for both aggressive acts and reaction from others in response to what might be perceived as provocation [51].

We observed an increase in police-recorded DV events with dementia among the older age groups for both POIs and victims. The plethora of evidence suggests that older individuals with dementia are at a high risk of abuse, especially in a carer setting [52,53]. Our findings add to this evidence base, showing that among victims aged 65 years and over, dementia was implicated in 47.33% (515/1088) of police-recorded DV events.

DV has been directly linked to severe mental illnesses including mood disorders [3]. Depression, in particular, has been associated with both victimization and perpetration of DV, with the extent of abuse corresponding with the severity of depression [54,55], something that has been reflected in our findings in the early and mid-adulthood groups [54].

Schizophrenia was proportionally higher among younger POIs (25-44 years old) and older victims (55-64 years old; 12.50%-14.70% [1188/9504-1346/9156] and 8.60% [99/1151], respectively). Our findings concur with previous studies suggesting that those with schizophrenia have a higher risk of perpetrating violence [56] and DV toward family members [57]. Previous studies also suggest that individuals with schizophrenia can be vulnerable in a domestic setting and open to experience more types of victimization [58,59].

In contrast to the police-recorded DV events with mental illness mentions identified using text mining, the structured data in the WebCOPS system contained a field entitled “mental illness related.” The total number of police-recorded DV events flagged as “mental illness related” was 1.03% (n=4295) of the total number of police-recorded DV events (N=416,441). This is in contrast to the number of police-recorded DV events that had extracted mental illness mentions from the narratives for the same events amounting to a total of 64,587 (15.51%). This discrepancy is likely explained by the police making a judgment call that mental illness was not considered as a factor for the cause of a DV event. However, through the application of text mining, we identified almost 16 times more police-recorded DV events with mental illness implicated than the police had classified in the fixed field as “mental illness related.” Further investigation to determine how these judgment calls are made by the police and the benefit of making this determination is required.

Automatically extracting mental illness mentions can add to existing data regarding POIs and victims involved in DV events and potentially in future events altering the police’s response toward a person with a known mental illness. One practical application of extracting this type of information can be its use in models along with other identified features (eg, abuse types, victim injuries) that could predict future offences within the area of DV and utilizing machine learning approaches, which could enable improved allocation of police sources for DV management. The successful implementation of text mining in police-recorded DV events may encourage greater use of unstructured data within law enforcement agencies that can be processed by such automated methodologies to extract important information regarding DV and other types of recorded offences (eg, sexual abuse, child neglect) with the police. The study has demonstrated that the trove of information contained in these events can be used to raise awareness among police officers regarding mental illness and, alongside better training, can improve the management of DV cases involving individuals with mental illness. With improved identification and awareness, it provides options for the police to divert individuals to hospital or community mental health services as appropriate. We believe this long-term preventative jurisprudence approach may provide opportunities to respond appropriately to mental illness in police-recorded DV events.

### Limitations

We cannot be certain that any individual extracted mention of a mental illness from police-recorded DV events is accurate. Police officers do undergo mental health training in 1- to 4-day courses (NSWPF, personal communication) so they can be aware if a POI or a victim may have a mental illness in addition to being informed by the victims and the POIs themselves, or potential witnesses of the event, and based on the evidence in the scene (eg, presence of medication prescriptions, drug and alcohol use). However, no literature exists on the validity of self-reported psychiatric status when shared with health professionals, or in other contexts where the data may be sought for administrative reasons, let alone with the police officers attending a home following a highly charged DV event. Studies reflecting self-diagnosed mental health conditions have demonstrated low validity with a substantial underreporting of mental health issues, which could be a reflected effort to avoid stigma [60,61].

Future work should examine the veracity of the police mentions of mental illness by using formal diagnostic information available from administrative data collections in hospital admissions, GP presentations, and community mental health services. It would be particularly interesting to see if mentions of diagnoses with high implied precision (ie, second- and particularly third-level diagnostic categories) are more likely to be validated by existing records than the more generic diagnoses. Such a study would allow a determination as to whether there is a bias in respect of greater police mentions of mental illness for POIs instead for the victims. In addition, further exploration will be conducted to investigate the observed differences between most commonly reported mental illnesses across the age groups of POIs and victims as well as to investigate whether the extracted information can be used as input toward predictive models for DV.

### Conclusions

This novel study involving the automated extraction of mental illness mentions through text mining from a large-scale data set of 416,441 police-recorded DV events provides potentially important information for mental health professionals and criminal justice policy makers to help address mental illness in police-recorded DV events. A trove of DV data are captured by the police as unstructured text that text mining can unearth. The information extracted from a large-scale set of police-recorded DV events suggests there may be more in-depth information related to trends in mental illness for victims and POIs. While this information can be seen as police insights in recorded DV events, it can provide the basis for examining the concordance of the extracted mental illness mentions with official diagnosis from health records and research that aims to assess the characteristics and features of victims and POIs involved in police-recorded DV events. This work will also explore whether extracted information can be used to design predictive models for those at risk of further victimization, to inform prevention strategies that could be implemented at the early stages of police involvement in a DV event.

## Appendix

Multimedia Appendix 1 A hypothetical example of a domestic violence event narrative as recorded by the NSWPF.

Multimedia Appendix 2 Mental illnesses listed in the ICD-10 including the eight new categories targeted for extraction in domestic violence events with examples as they appeared in the police event narratives.

Multimedia Appendix 3 The ICD-10 Mental and Behavioural Disorders schema used to map the extracted mental illness mentions containing three levels (first, second and third).

Multimedia Appendix 4 Percentage of domestic violence events involving POIs with the top ten most commonly mentioned mental illnesses at ICD-10 level 2 across age groups.

Multimedia Appendix 5 Percentage of domestic violence events involving victims with the top ten most commonly mentioned mental illnesses at ICD-10 level 2 across age groups.

## Abbreviations

DV: domestic violence
GATE: General Architecture for Text Engineering
ICD: International Classification of Diseases
NSWPF: New South Wales Police Force
POI: persons of interest
