# Supplementary figures and images for: Prevalence of Mental Illnesses in Domestic Violence Police Records: Text Mining Study
Source: J Med Internet Res. 2020 Dec 24;22(12):e23725. doi: 10.2196/23725 (PMC7790609; doi:10.2196/23725)

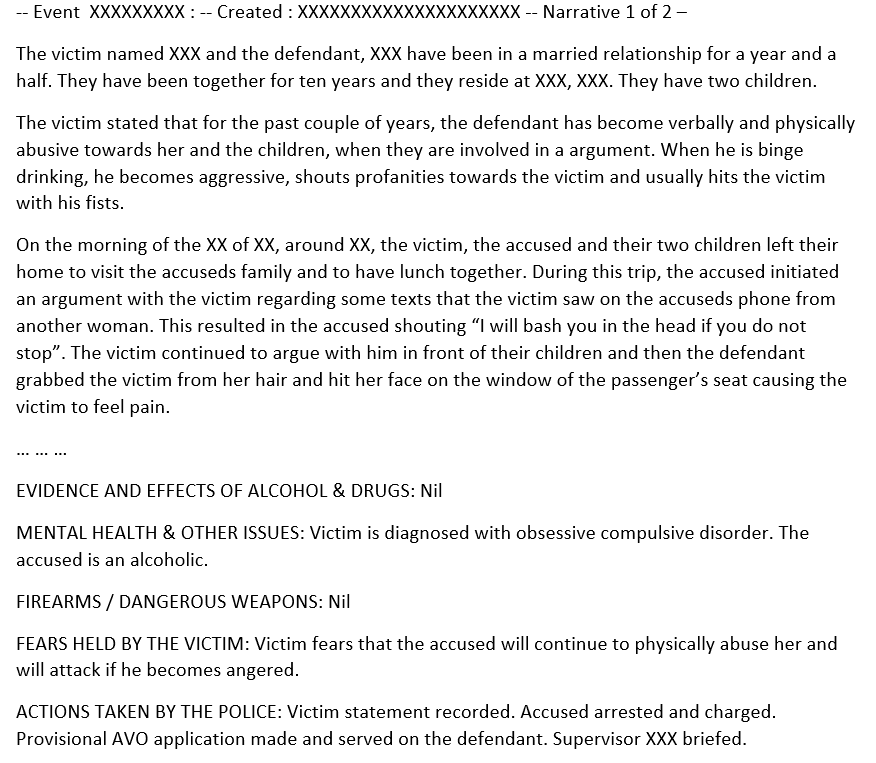

Supplement: Multimedia Appendix 1 [file jmir_v22i12e23725_app1.docx]
