# Supplementary material for: Prevalence of Mental Illnesses in Domestic Violence Police Records: Text Mining Study
Source: J Med Internet Res. 2020 Dec 24;22(12):e23725. doi: 10.2196/23725 (PMC7790609; doi:10.2196/23725)
Supplement: Multimedia Appendix 2 [file jmir_v22i12e23725_app2.docx]

| **ICD-10 Mental health condition** | **Examples of mental illness mentions in police narratives** |
| --- | --- |
| 1. Mental disorders due to known physiological conditions | vascular dementia, unspecified dementia |
| 1. Mental and behavioural disorders due to psychoactive substance abuse | alcohol related disorders, cannabis addiction, nicotine dependence |
| 1. Schizophrenia, schizotypal, delusional and other non-mood psychotic disorders | schizophrenia, delusions, schizoaffective disorder |
| 1. Mood [affective] disorders | manic episodes, bipolar disorder, depression |
| 1. Anxiety, dissociative, stress-related, somatoform and other nonpsychotic mental disorders | phobia, dissociative disorder, body dysmorphic disorder |
| 1. Behavioural syndromes associated with physiological disturbances and physical factors | eating disorders, bulimia, anorexia |
| 1. Disorders of adult personality and behaviour | paranoid personality disorder, borderline personality disorder, kleptomania |
| 1. Intellectual disabilities | intellectual disability, severe intellectual disability |
| 1. Pervasive and specific developmental disorders | autism, mathematics disorder, phonological disorder |
| 1. Behavioural and emotional disorders with onset usually occurring in childhood and adolescence | attention deficit hyperactivity disorder, antisocial personality disorder, transient tic disorder |
| 1. Unspecified mental disorder | mental health issues, mental condition, mental health problem |
| 1. Intentional self-harm | self-harm, cut herself on purpose, self-harming issues |
| 1. Injury of unspecified body region | suicide attempt, multiple suicide attempt, attempted to kill himself |
| 1. Symptoms, signs and abnormal clinical and laboratory findings | suicidal ideation, suicidal thoughts, suicidal tendencies |
| 1. Diseases of the nervous system | Alzheimer’s disease, Huntington’s disease, frontotemporal dementia |
| 1. Unspecified diseases of the nervous system | neurological disorder, other neurological disorder |
| 1. Systematic atrophies primarily affecting the central nervous system | Huntington’s disease, Huntingtons |
| 1. Chromosomal abnormalities, not elsewhere classified | Down syndrome |
| 1. Drug prescription abuse | addiction in prescribed medications, abusing prescribed meds |
| 1. Traumatic brain injury | brain damage, serious brain injury, brain trauma |
| 1. Substance abuse | substance abuse problem, ongoing drug abuse problems |
| 1. Mental health medications antipsychotics | Clozapine, anti psychotic medications, Risperdal |
| 1. Mental health medications neuroleptics | neuroleptic medications, neuroleptic drugs |
| 1. Mental health medications anti-anxiety | Xanax, medications: anxiety, Alprazolam |
| 1. Mental health medications antidepressants | Escitalopram, Anafranil, anti-depressant medication |
| 1. Unspecified drug induced disorders | drug induced disorder, drug induced mental health problem |
