# Supplementary material for: Prevalence of Mental Illnesses in Domestic Violence Police Records: Text Mining Study
Source: J Med Internet Res. 2020 Dec 24;22(12):e23725. doi: 10.2196/23725 (PMC7790609; doi:10.2196/23725)
Supplement: Multimedia Appendix 3 [file jmir_v22i12e23725_app3.docx]

| **First level** | **Second level** | **Third level** | **Fourth level** |
| --- | --- | --- | --- |
| Mental disorders due to known physiological conditions | Vascular dementia | - | - |
|  | Unspecified dementia | - | - |
|  | Delirium | - | - |
|  | Unspecified mental disorder due to known physiological condition | - | - |
| Mental and behavioral disorders due to psychoactive substance use | Alcohol related disorders | - | - |
|  | Opioid related disorders | - | - |
|  | Cannabis related disorders | - | - |
|  | Cocaine related disorders | - | - |
|  | Other stimulant related disorders | - | - |
|  | Nicotine dependence | - | - |
|  | Other psychoactive substance related disorders | - | - |
| Schizophrenia, schizotypal, delusional, and other non-mood psychotic disorders | Schizophrenia | Paranoid schizophrenia | - |
|  |  | Disorganized schizophrenia | - |
|  |  | Catatonic schizophrenia | - |
|  |  | Undifferentiated schizophrenia | - |
|  |  | Residual schizophrenia | - |
|  |  | Other schizophrenia | - |
|  |  | Unspecified schizophrenia | - |
|  | Schizotypal disorder | - | - |
|  | Delusional disorders | - | - |
|  | Brief psychotic disorder | - | - |
|  | Shared psychotic disorder | - | - |
|  | Schizoaffective | - | - |
|  | Unspecified psychosis not due to a substance or known physiological condition | - | - |
| Mood [affective] disorders | Manic episode | - | - |
|  | Bipolar disorder | Bipolar disorder, unspecified | - |
|  |  | Other bipolar disorders | Bipolar II disorders |
|  | Major depressive disorder, single episode | Postpartum depression | - |
|  | Major depressive disorder, recurrent | Other recurrent depressive disorders |  |
|  | Persistent mood disorders | Cyclothymic disorder | - |
|  |  | Dysthymic disorder | - |
|  |  | Other persistent mood disorders | Disruptive mood dysregulation disorder |
|  | Unspecified mood disorder | - | - |
| Anxiety, dissociative, stress-related, somatoform and other nonpsychotic mental disorders | Phobic anxiety disorder | Agoraphobia | - |
|  |  | Social phobias | Social phobia, generalised |
|  |  | Specific isolated phobias | Arachnophobia |
|  |  |  | Claustrophobia |
|  |  |  | Acrophobia |
|  |  |  | Androphobia |
|  |  |  | Gynaecophobia |
|  |  | Other phobic anxiety disorders | - |
|  |  | Phobic anxiety disorder, unspecified | - |
|  | Other anxiety disorders | Panic disorder | - |
|  |  | Generalised anxiety disorder | - |
|  |  | Anxiety disorder, unspecified | - |
|  | Obsessive compulsive disorders | Hoarding disorder | - |
|  |  | Excoriation disorder | - |
|  |  | Obsessive compulsive disorder, unspecified | - |
|  | Reaction to severe stress and adjustment disorders | Acute stress reaction | - |
|  |  | Post-traumatic stress disorder | - |
|  |  | Adjustment disorders | - |
|  | Dissociative and conversion disorders | Dissociative amnesia | - |
|  |  | Dissociative fugue | - |
|  |  | Dissociative stupor | - |
|  |  | Other dissociative and conversion disorders | Dissociative identity disorder |
|  |  | Dissociative and conversion disorder, unspecified | - |
|  | Somatoform disorders | Somatization disorder | - |
|  |  | Undifferentiated somatoform disorder | - |
|  |  | Hypochondrial disorders | Body dysmorphic disorder |
|  |  |  | Hypochondriasis |
|  | Other nonpsychotic mental disorders | Depersonalization-derealization syndrome | - |
|  |  | Pseudobulbar affect | - |
|  |  | Nonpsychotic mental disorder, unspecified | - |
| Behavioral syndromes associated with physiological disturbances and physical factors | Eating disorders | Anorexia nervosa | - |
|  |  | Bulimia nervosa | - |
|  |  | Other eating disorders | Binge eating disorder |
|  |  |  | Avoidant food intake disorder |
|  | Sleep disorders not due to a substance or known physiological condition | Insomnia not due to a substance or known physiological condition | Primary insomnia |
|  |  |  | Adjustment insomnia |
|  |  |  | Paradoxical insomnia |
|  |  |  | Psychophysiologic insomnia |
|  | Sexual dysfunction not due to a substance or known physiological condition | Hypoactive sexual desire disorder | - |
|  | Abuse of non-psychoactive substances | Abuse of steroids or hormones | - |
| Disorders of adult personality and behavior | Specific personality disorders | Paranoid personality disorder | - |
|  |  | Schizoid personality disorder | - |
|  |  | Antisocial personality disorder | - |
|  |  | Borderline personality disorder | - |
|  |  | Histrionic personality disorder | - |
|  |  | Obsessive compulsive personality disorder | - |
|  |  | Avoidant personality disorder | - |
|  |  | Dependent personality disorder | - |
|  |  | Other specific personality disorders | Narcissistic personality disorder |
|  |  | Personality disorder, unspecified | - |
|  | Impulse disorders | Pathological gambling | - |
|  |  | Pyromania | - |
|  |  | Kleptomania | - |
|  |  | Trichotillomania | - |
|  |  | Other impulse disorders | Intermittent explosive disorder |
|  |  | Impulse disorder, unspecified | - |
|  | Gender identity disorders | Transsexualism | - |
|  |  | Dual role transsexualism | - |
|  |  | Gender identify disorder | - |
|  | Paraphilias | Fetishism | - |
|  |  | Transvestic fetishism | - |
|  |  | Exhibitionism | - |
|  |  | Voyeurism | - |
|  |  | Paedophilia | - |
|  |  | Sadomasochism | - |
|  |  | Other paraphilias | Frotteurism |
|  | Other personalities of adult and personality behaviour | Factitious disorder | - |
|  | Unspecified disorder of adult personality and behavior | - | - |
| Intellectual disabilities | Mild intellectual disabilities | - | - |
|  | Moderate intellectual disabilities | - | - |
|  | Severe intellectual disabilities | - | - |
|  | Profound intellectual disabilities | - | - |
|  | Unspecified intellectual disabilities | - | - |
| Pervasive and specific developmental disorders | Specific developmental disorders of speech and language | Phonological disorder | - |
|  |  | Expressive language disorder | - |
|  |  | Mixed receptive-expressive language disorder | - |
|  |  | Other developmental disorders of speech and language | Childhood onset fluency disorder |
|  |  |  | Social pragmatic communication disorder |
|  | Specific developmental disorders of scholastic skills | Specific reading disorder | - |
|  |  | Mathematics disorder | - |
|  |  | Other developmental disorders of scholastic skills | Disorder of written expression |
|  | Pervasive developmental disorders | Autism | - |
|  |  | Rett’s syndrome | - |
|  |  | Asperger’s syndrome | - |
|  |  | Pervasive developmental disorder, unspecified | - |
|  | Unspecified disorder of psychological development | - | - |
| Behavioral and emotional disorders with onset usually occurring in childhood and adolescence | Attention-deficit hyperactivity disorders | - | - |
|  | Conduct disorders | Conduct disorder, unspecified | - |
|  |  | Oppositional defiant disorder | - |
|  | Emotional disorders with onset specific to childhood | Separation anxiety disorder of childhood | - |
|  | Disorders of social functioning with onset specific to childhood and adolescence | Selective mutism | - |
|  |  | Reactive attachment disorder of childhood | - |
|  |  | Disinhibited attachment disorder of childhood | - |
|  | Tic disorder | Transient tic disorder | - |
|  |  | Chronic motor or vocal tic disorder | - |
|  |  | Tourette's disorder | - |
|  | Other behavioural and emotional disorders | Unspecified behavioural and emotional disorders | - |
| Unspecified mental disorder |  |  | - |
| Other degenerative diseases of the nervous system | Alzheimer’s disease | Alzheimer’s disease, unspecified | - |
|  | Other degenerative diseases of the nervous system, not elsewhere classified | Frontotemporal dementia | - |
| Systemic atrophies primarily affecting the central nervous system | Huntington’s disease | - | - |
| Injury of unspecified body region | Injury of unspecified body region | Unspecified injury | Suicide attempt |
| Symptoms and signs involving cognition, perception, emotional state and behavior | Symptoms and signs involving emotional state | Other symptoms and signs involving emotional state | Homicidal and suicidal ideations |
| Chromosomal abnormalities, not elsewhere classified | Down syndrome | Down syndrome, unspecified | - |
| Intentional self-harm | - | - | - |
| Unspecified diseases of the nervous system | - | - | - |
| Unspecified drug induced disorders | - | - | - |
| Medications - neuroleptics | - | - | - |
| Medications - antipsychotics | - | - | - |
| Medications – anti anxiety | - | - | - |
| Medications – antidepressants | - | - | - |
| Traumatic brain injury | - | - | - |
| Substance abuse | - | - | - |
| Drug prescription abuse | - | - | - |

**Table 3:** Examples of extracted mental illness mentions (including misspellings) mapped into the expanded ICD-10 schema. Note the inclusion of extra defined categories such as ‘Medications – antidepressants’.

| **Extracted mental illness mention** | **Mapped mental illness (ICD-10)** | **1^st^ level (ICD-10)** | **2^nd^ level (ICD-10)** | **3^rd^ level (ICD-10)** |
| --- | --- | --- | --- | --- |
| Self-harming issues | Self-harm | Intentional self-harm | - | - |
| Scitzophrenia | Schizophrenia | Schizophrenia, schizotypal, delusional, and other non-mood psychotic disorder | Schizophrenia | Schizophrenia, unspecified |
| Schizotypal disorder | Schizotypal disorder | Schizophrenia, schizotypal, delusional, and other non-mood psychotic disorder | Schizotypal disorder | - |
| Mental health issues | Unspecified mental illness | Unspecified mental illness | - | - |
| Zoloft | Zoloft | Medications - antidepressants | - | - |
| Intermittent explosive disorder | Intermittent explosive disorder | Disorders of adult personality and behavior | Impulse disorders | Intermittent explosive disorder* |

*Note that ‘intermittent explosive disorder’ is a fourth level ICD-10 classification but for reporting purposes we included this disorder at the third level.

**Table 4:** Percentage of domestic violence events involving POIs with the top ten most commonly mentioned mental illnesses at ICD-10 level 2 across age groups.

|  | | **age groups** | | | | | | | |
| --- | --- | --- | --- | --- | --- | --- | --- | --- | --- |
|  |  | 0-14 | 15-24 | 25-34 | 35-44 | 45-54 | 55-64 | 65+ |  |
| **mental illness** | **dementia, unspecified** | 0.1 | 0.0 | 0.0 | 0.0 | 0.3 | 1.4 | 25.9 |  |
|  | **schizophrenia** | 0.4 | 4.4 | 12.5 | 14.7 | 11.0 | 9.0 | 3.7 |  |
|  | **bipolar disorder** | 1.8 | 7.3 | 13.0 | 14.4 | 13.1 | 11.5 | 5.6 |  |
|  | **intellectual disability, unspecified** | 2.9 | 3.2 | 3.0 | 1.5 | 1.6 | 1.8 | 2.3 |  |
|  | **other anxiety disorders** | 5.6 | 6.7 | 5.7 | 4.7 | 4.3 | 4.0 | 2.5 |  |
|  | **major depressive disorder, single episode** | 5.6 | 14.8 | 20.3 | 20.6 | 20.5 | 21.2 | 14.5 |  |
|  | **conduct disorders** | 11.8 | 5.6 | 0.6 | 0.2 | 0.2 | 0.3 | 0.4 |  |
|  | **pervasive developmental disorder** | 14.2 | 9.2 | 2.5 | 0.8 | 0.4 | 0.5 | 0.3 |  |
|  | **other behavioural and emotional disorders with onset usually occurring in childhood and adolescence** | 18.2 | 15.8 | 10.7 | 7.7 | 7.8 | 6.4 | 4.2 |  |
|  | **attention deficit hyperactivity disorder** | 28.1 | 19.6 | 6.5 | 2.6 | 2.4 | 1.9 | 1.7 |  |
|  | **alcohol abuse** | 0.0 | 1.4 | 7.7 | 16.4 | 23.6 | 29.7 | 23.2 |  |

* Denominators for percentages were total number of events with a recorded mental illness for that age group.

**Table 5:** Percentage of domestic violence events involving victims with the top ten most commonly mentioned mental illnesses at ICD-10 level 2 across age groups.

|  | | **age groups** | | | | | | |
| --- | --- | --- | --- | --- | --- | --- | --- | --- |
|  |  | **0-14** | **15-24** | **25-34** | **35-44** | **45-54** | **55-64** | **65+** |
| **mental illness** | **obsessive-compulsive disorder** | 1.6 | 0.0 | 0.0 | 0.0 | 0.0 | 0.0 | 0.0 |
|  | **schizophrenia** | 2.1 | 2.5 | 5.4 | 6.5 | 7.6 | 8.6 | 4.4 |
|  | **intellectual disability, mild** | 3.3 | 0.0 | 0.0 | 0.0 | 0.0 | 0.0 | 0.0 |
|  | **intellectual disability, unspecified** | 5.0 | 8.2 | 6.9 | 3.5 | 3.6 | 6.3 | 3.3 |
|  | **conduct disorders** | 5.8 | 0.0 | 0.0 | 0.0 | 0.0 | 0.0 | 0.0 |
|  | **other anxiety disorders** | 6.7 | 13.3 | 14.8 | 13.2 | 10.9 | 10.1 | 4.4 |
|  | **major depressive disorder, single episode** | 7.5 | 21.3 | 25.9 | 26.3 | 21.5 | 19.5 | 8.7 |
|  | **other behavioural and emotional disorders with onset usually occurring in childhood and adolescence** | 8.3 | 7.8 | 5.0 | 4.9 | 5.0 | 3.6 | 1.9 |
|  | **pervasive developmental disorder** | 23.8 | 7.5 | 0.0 | 0.0 | 2.3 | 0.0 | 0.0 |
|  | **attention deficit hyperactivity disorder** | 29.2 | 15.3 | 7.7 | 6.4 | 6.9 | 5.2 | 3.4 |
|  | **specific personality disorders** | 0.0 | 2 | 3.4 | 3.3 | 0.0 | 2.7 | 0.0 |
|  | **reaction to severe stress, and adjustment disorder** | 0.0 | 2.4 | 2.6 | 2.9 | 3.6 | 0.0 | 0.0 |
|  | **bipolar disorder** | 0.0 | 7.4 | 12.6 | 13.1 | 12.6 | 10.1 | 4.5 |
|  | **alcohol abuse** | 0.0 | 0 | 3.8 | 9.6 | 13.9 | 18.3 | 6.6 |
|  | **dementia, unspecified** | 0.0 | 0 | 0 | 0 | 0 | 2.2 | 47.3 |
|  | **Alzheimer’s disease** | 0.0 | 0 | 0 | 0 | 0 | 0 | 4 |

* Denominators for percentages were total number of events with a recorded mental illness for that age group.
