# Supplementary material for: Prevalence of Mental Illnesses in Domestic Violence Police Records: Text Mining Study
Source: J Med Internet Res. 2020 Dec 24;22(12):e23725. doi: 10.2196/23725 (PMC7790609; doi:10.2196/23725)
Supplement: Multimedia Appendix 4 [file jmir_v22i12e23725_app4.docx]

**Table 4:** Percentage of domestic violence events involving POIs with the top ten most commonly mentioned mental illnesses at ICD-10 level 2 across age groups.

|  | | **age groups** | | | | | | | |
| --- | --- | --- | --- | --- | --- | --- | --- | --- | --- |
|  |  | 0-14 | 15-24 | 25-34 | 35-44 | 45-54 | 55-64 | 65+ |  |
| **mental illness** | **dementia, unspecified** | 0.1 | 0.0 | 0.0 | 0.0 | 0.3 | 1.4 | 25.9 |  |
|  | **schizophrenia** | 0.4 | 4.4 | 12.5 | 14.7 | 11.0 | 9.0 | 3.7 |  |
|  | **bipolar disorder** | 1.8 | 7.3 | 13.0 | 14.4 | 13.1 | 11.5 | 5.6 |  |
|  | **intellectual disability, unspecified** | 2.9 | 3.2 | 3.0 | 1.5 | 1.6 | 1.8 | 2.3 |  |
|  | **other anxiety disorders** | 5.6 | 6.7 | 5.7 | 4.7 | 4.3 | 4.0 | 2.5 |  |
|  | **major depressive disorder, single episode** | 5.6 | 14.8 | 20.3 | 20.6 | 20.5 | 21.2 | 14.5 |  |
|  | **conduct disorders** | 11.8 | 5.6 | 0.6 | 0.2 | 0.2 | 0.3 | 0.4 |  |
|  | **pervasive developmental disorder** | 14.2 | 9.2 | 2.5 | 0.8 | 0.4 | 0.5 | 0.3 |  |
|  | **other behavioural and emotional disorders with onset usually occurring in childhood and adolescence** | 18.2 | 15.8 | 10.7 | 7.7 | 7.8 | 6.4 | 4.2 |  |
|  | **attention deficit hyperactivity disorder** | 28.1 | 19.6 | 6.5 | 2.6 | 2.4 | 1.9 | 1.7 |  |
|  | **alcohol abuse** | 0.0 | 1.4 | 7.7 | 16.4 | 23.6 | 29.7 | 23.2 |  |

* Denominators for percentages were total number of events with a recorded mental illness for that age group.
