# Supplementary material for: Prevalence of Mental Illnesses in Domestic Violence Police Records: Text Mining Study
Source: J Med Internet Res. 2020 Dec 24;22(12):e23725. doi: 10.2196/23725 (PMC7790609; doi:10.2196/23725)
Supplement: Multimedia Appendix 5 [file jmir_v22i12e23725_app5.docx]

**Table 5:** Percentage of domestic violence events involving victims with the top ten most commonly mentioned mental illnesses at ICD-10 level 2 across age groups.

|  | | **age groups** | | | | | | |
| --- | --- | --- | --- | --- | --- | --- | --- | --- |
|  |  | **0-14** | **15-24** | **25-34** | **35-44** | **45-54** | **55-64** | **65+** |
| **mental illness** | **obsessive-compulsive disorder** | 1.6 | 0.0 | 0.0 | 0.0 | 0.0 | 0.0 | 0.0 |
|  | **schizophrenia** | 2.1 | 2.5 | 5.4 | 6.5 | 7.6 | 8.6 | 4.4 |
|  | **intellectual disability, mild** | 3.3 | 0.0 | 0.0 | 0.0 | 0.0 | 0.0 | 0.0 |
|  | **intellectual disability, unspecified** | 5.0 | 8.2 | 6.9 | 3.5 | 3.6 | 6.3 | 3.3 |
|  | **conduct disorders** | 5.8 | 0.0 | 0.0 | 0.0 | 0.0 | 0.0 | 0.0 |
|  | **other anxiety disorders** | 6.7 | 13.3 | 14.8 | 13.2 | 10.9 | 10.1 | 4.4 |
|  | **major depressive disorder, single episode** | 7.5 | 21.3 | 25.9 | 26.3 | 21.5 | 19.5 | 8.7 |
|  | **other behavioural and emotional disorders with onset usually occurring in childhood and adolescence** | 8.3 | 7.8 | 5.0 | 4.9 | 5.0 | 3.6 | 1.9 |
|  | **pervasive developmental disorder** | 23.8 | 7.5 | 0.0 | 0.0 | 2.3 | 0.0 | 0.0 |
|  | **attention deficit hyperactivity disorder** | 29.2 | 15.3 | 7.7 | 6.4 | 6.9 | 5.2 | 3.4 |
|  | **specific personality disorders** | 0.0 | 2 | 3.4 | 3.3 | 0.0 | 2.7 | 0.0 |
|  | **reaction to severe stress, and adjustment disorder** | 0.0 | 2.4 | 2.6 | 2.9 | 3.6 | 0.0 | 0.0 |
|  | **bipolar disorder** | 0.0 | 7.4 | 12.6 | 13.1 | 12.6 | 10.1 | 4.5 |
|  | **alcohol abuse** | 0.0 | 0 | 3.8 | 9.6 | 13.9 | 18.3 | 6.6 |
|  | **dementia, unspecified** | 0.0 | 0 | 0 | 0 | 0 | 2.2 | 47.3 |
|  | **Alzheimer’s disease** | 0.0 | 0 | 0 | 0 | 0 | 0 | 4 |

* Denominators for percentages were total number of events with a recorded mental illness for that age group.
